# Mechanosensitivity during straight leg raise and slump neurodynamic tests in people with type 1 diabetes mellitus and diabetic peripheral neuropathy

**DOI:** 10.1080/10669817.2025.2544285

**Published:** 2025-08-12

**Authors:** Georgia Koutsoflini, Antonios Lepouras, Colette Ridehalgh

**Affiliations:** a School of Sports and Health Sciences, University of Brighton, Eastbourne, UK; b Diabetes Department, Metropolitan General Hospital, Athens, Greece

**Keywords:** Diabetic peripheral neuropathy, type 1 diabetes, nerve mechanosensitivity, lower limb neurodynamic tests, straight leg raise test, slump test

## Abstract

**Objectives:**

Neurodynamic tests are clinical tests used to identify heightened nerve mechanosensitivity but may be negative in the presence of severe neuropathy, as seen in people with carpal tunnel syndrome and type 2 diabetes. It is not known if this also occurs in people with diabetic peripheral neuropathy (DPN) from type 1 diabetes mellitus (T1DM). The primary aim of this study is to determine the proportion of positive neurodynamic tests in people with T1DM and DPN. The secondary aim is to assess whether the severity of DPN influences the presence of a positive neurodynamic test.

**Methods:**

This is a cross-sectional study. Forty-three participants with T1DM and DPN were assessed using straight leg raise (SLR) and slump neurodynamic tests to determine a positive and negative test. DPN severity was graded according to Toronto Clinical Scoring System (TCSS).

**Results:**

Forty-six percent and 56% of participants had positive SLR and slump tests, respectively, indicating heightened nerve mechanosensitivity. There was a statistically significant association between negative neurodynamic tests and DPN severity (*p* < 0.0001). In addition, participants with negative neurodynamic tests had significantly higher TCSS scores compared to participants with positive neurodynamic tests (*p* < 0.0001).

**Discussion/Conclusion:**

People with T1DM and severe DPN, as graded by TCSS, are more likely to demonstrate negative neurodynamic tests than those with mild DPN. Future studies should investigate the relationship between neurodynamic tests and nerve function in other conditions. This supports previous research on negative neurodynamic tests in severe neuropathy, suggesting that neurodynamic tests should not be used alone to determine nerve involvement.

## 1. Introduction

Type 1 diabetes mellitus (T1DM) is a chronic metabolic disorder characterized by absolute insulin deficiency [1]. It is common in children and young adults, affecting 10% of the general population worldwide [2]. Insulin deficiency results in elevated glucose levels, a condition termed hyperglycemia [1]. Poor diabetic control and prolonged hyperglycemia severely impact peripheral nerve function, resulting in diabetic peripheral neuropathy (DPN) [3]. DPN has been defined as ‘the presence of symptoms and/or signs of peripheral nerve dysfunction in people with diabetes after exclusion of other causes’ [4] as cited in [5, p.1458] and is likely to affect 50% of people with T1DM [6]. Clinical presentation of DPN includes bilateral, symmetrical pain, and/or numbness and/or paresthesia that initially occur distally in toes. These symptoms follow a ‘stocking and glove’ distribution and, as the disease progresses, are apparent not only in lower but also in upper limbs [7].

The exact pathophysiological mechanism of DPN is not clear [8]. Metabolic imbalance within nerves of people with diabetes, caused by excess glucose gives rise to nerve inflammation, ischemia, and mitochondrial dysfunction [8]. Thus, gradual nerve demyelination, degeneration, axonal atrophy and eventually nerve cell death occurs [8].

Severity of DPN is most accurately determined by nerve and skin biopsy [9,10]. Nerve conduction studies and quantitative sensory testing are alternative diagnostic methods vital for a confirmed DPN diagnosis [3]. However, many of these techniques are not available in routine clinical practice [9,10], thus DPN screening tools, such as the Toronto Clinical Scoring System (TCSS), have been developed. The total TCSS score is derived from a combination of DPN symptoms, signs and neurological examination findings which identifies the presence and severity of DPN [11].

Nerve mechanosensitivity is a normal response of the nervous system to excessive mechanical loading during lower limb movements [12–14] Injury or inflammation may further mechanically sensitize nerve fibers leading to heightened nerve mechanosensitivity [15,16]. One way to assess such heightened nerve mechanosensitivity is with the use of neurodynamic tests. Lower limb neurodynamic assessment, such as straight leg raise test (SLR) and slump test, are clinical procedures used to identify heightened nerve mechanosensitivity of the lumbosacral roots, along with their distal continuations into the sciatic nerve, during a series of lower limb movements [17,18]. Heightened nerve mechanosensitivity response has been identified in a few neuropathic pain conditions such as carpal tunnel syndrome [19] and lumbar radiculopathy [20,21].

In order for a neurodynamic test to be deemed positive, the patient’s symptoms have to be reproduced and change when a joint distal to the area of symptoms is moved (structural differentiation) [13,22,23]. This identifies nerve tissue as a likely contributor of the individual’s symptoms since structural differentiation alters the mechanical load applied to the nerve with minimal effect on the other local tissue sources [17,22,24].

Whilst a positive neurodynamic test suggests heightened nerve mechanosensitivity [17], a negative test has been proposed to enable the clinician to rule neuropathy less likely [21,25]. However, it has been reported that a significant proportion of people with confirmed severe neuropathy, including lumbar radiculopathy (58%) [26] and carpal tunnel syndrome (up to 72%) [19,27], demonstrate negative neurodynamic tests. In addition, a similar trend has been seen in individuals with severe DPN from type 2 diabetes mellitus during SLR test [28].

Since patients with T1DM may develop DPN [3] and clinicians may use neurodynamic tests to suggest heightened nerve mechanosensitivity, it is important to explore lower limb neurodynamic tests in this population. This will enhance the understanding and interpretation of lower limb neurodynamic tests in people with DPN.

The aims of this study are:

1. To establish the proportion of people with T1DM and DPN with positive SLR and slump tests.
2. To establish if the severity of DPN influences the result of SLR and slump tests.

## 2. Methods

### 2.1. Ethical approval

The study was conducted in accordance with the Helsinki Declaration and was approved by the Ethics and Governance Research Committee of the host University in the United Kingdom. In addition, the scientific committee of the hospital involved reviewed the study and confirmed that additional ethical approval by them was not necessary.

### 2.2. Study design

This is a cross-sectional study which took place in the diabetes department of a private hospital in Athens, Greece.

### 2.3. Sample size calculation

In this study, TCSS scores were compared between participants with a positive and negative SLR test and between those with a positive and negative slump test. Sample size calculation was based on previously published data in a varied cohort of patients with neuropathic pain using the TCSS to grade the presence or absence of DPN [29] and revealed that 34 participants were needed per group (positive/negative neurodynamic test) to identify significant differences in TCSS between groups with 80% power and significance set at *p* = .05 (effect size 0.78).

### 2.4. Recruitment

Participants were recruited from the outpatient list of the diabetes department of the private hospital through invitation e-mails and online invitation posts. A consultant, who specialized in diabetes, invited patients during their consultation and, based on patient interest, screened for exclusion criteria. Moreover, invitation e-mails were sent to all patients with T1DM whose details appeared on the department’s outpatient list. Social media was used to distribute online invitation posts in diabetes-related groups.

### 2.5. Participants

Forty-three participants (22 male and 21 female), aged between 18–64 years old (mean = 38.2 years), met the inclusion criteria of 10 years or more of T1DM duration and presence of DPN related symptoms such as bilateral symmetrical foot and/or lower limb pain and/or paresthesia and/or numbness. Participants either already had a confirmed DPN diagnosis via nerve conduction studies [3] or scored a minimum of 6 points on TCSS which corresponds to mild DPN [11].

Participants were excluded if they had severe feet deformities, infections, open ulcers and fissures, subclinical DPN, had severe and irritable symptoms which meant they could not tolerate the neurodynamic tests being applied, had other lower limb or lower back conditions e.g. sciatica, had other significant medical conditions or were pregnant.

All participants gave both written and verbal informed consent before commencing the study.

### 2.6. Research procedure

The assessment procedure lasted up to 1 hour and 30 minutes for each participant. It consisted of DPN examination and nerve mechanosensitivity assessment. All procedures were carried out by a Musculoskeletal Physiotherapist with 8 years of clinical experience. The spine and lower limbs were assessed to rule out participants with musculoskeletal conditions in the lower spine and lower limbs. Additionally, a consultant who specializes in diabetes assessed the participants and ensured that no fissures or ulcers were present in the feet.

#### 2.6.1. Neuropathy assessment

The TCSS was used to assess and grade DPN severity. It has shown moderate significant negative correlation with myelinated nerve fiber density (*r* = −0.479, *p* < 0.0001) [11], indicating that as myelinated nerve fibers degenerate the TCSS score increases in people with DPN. The TCSS groups individuals using an ordinal scale. The highest possible score being 19 points. DPN is graded as ‘severe’ when the individual scores >13 points, as ‘moderate’ when the individual scores between 9 and 12 points and as ‘mild’ when the individual scores between 6 and 8 points. A TCSS score of less than 6 points, indicates ‘no neuropathy’ [11].

The clinical DPN screening tool descriptively assesses symptom characteristics and physically examines reflex and sensory function. Participants were asked to describe the presence or absence of neuropathic related symptoms such as pain, weakness, numbness, and paresthesia in the lower and upper limbs. Additionally, the presence or absence of gait related disturbances, such as unsteadiness, were asked. The presence of each symptom corresponded to one point, whereas the absence of a symptom is scored as zero [11].

The clinical examination included sensory testing of small and large nerve fibers on the dorsum of participant’s great toe [11]. The chest area was used as a reference testing site since both lower limbs are commonly affected in DPN [7]. The researcher used a neurotip and glass tubes with lukewarm and cold water [30] to assess pinprick and warm/cold sensation, respectively. In addition, vibration sensation was examined by a 128 Hz tuning fork. A cotton wool ball was used to assess light touch sensation. Participants closed their eyes during the examination. A score of 1 point was given when a participant felt a reduction in a sensory stimulus. Sensations similar to the chest area (reference testing site) were assigned a score of 0. When testing for joint position sense, failing to identify the direction of big toe movement (upwards or downwards), was awarded 1 point while correct answers received 0 point. Knee and ankle reflexes were examined in both lower limbs using a reflex hammer. A reduction in the reflex received 1 point whilst an absent reflex received 2 points. Normal reflexes were scored as 0 [11].

#### 2.6.2. Heightened nerve mechanosensitivity assessment

Immediately after completing the TCSS, nerve mechanosensitivity was assessed with SLR and slump tests in a random order. In order to randomize the test procedure, 2 identical pieces of paper with ‘SLR test’ and ‘slump test’ written on them were folded to hide the test name and were presented to each participant. Following that, each participant was instructed to pick one of the folded pieces of paper. The participant’s choice would determine which of the 2 tests would be performed first. The test written on the remaining piece of paper would be performed second.

The participant laid supine on the plinth for the SLR test [31]. Depending on the area of the participant’s symptoms, a different ankle sensitizing maneuver was applied in an attempt to sensitize the test to the specific nerve of the lower extremity (e.g. plantarflexion and inversion if anterolateral lower leg/foot symptoms for common peroneal nerve [32]. The researcher moved the participant’s hip into flexion with knee extension while the ankle was positioned into dorsiflexion and plantarflexion and inversion as determined by the area of the participant’s symptoms. When symptoms were reproduced in the ankle, the hip joint was used as the structural differentiation maneuver. When symptoms were reproduced on the posterior thigh area, the ankle was used as the structural differentiation maneuver [33,34]. In addition, if no symptoms were reproduced with foot, ankle or hip flexion movements, additional movements to the hip (adduction and/or medial rotation) were used to further challenge the sciatic nerve and its extensions [31,35]. The test was considered positive when the participant’s area of symptoms was reproduced and changed in accordance with the structural differentiation maneuver [23].

A similar procedure followed for the slump test. In a sitting position, participants were asked to hold their hands behind back, flex the trunk, flex the cervical spine, move their ankle into dorsiflexion (or plantarflexion/inversion) and extend the knee [31]. As soon as the participant’s symptoms were reproduced, they were asked to stop extending their knee and, at this point, were asked by the researcher to look up toward the ceiling [31]. Hence, cervical extension was chosen as structural differentiation maneuver [31]. A change in the severity of the participant’s symptoms following cervical extension indicated a positive test [23]. If the participant’s symptoms remained unchanged following cervical extension, the slump test was considered negative [23].

If the participant’s symptoms were not reproduced at all during either neurodynamic assessment, the test was considered negative.

### 2.7. Statistical analysis

IBM SPSS statistics version 25 and PRISM (GraphPad Software, Boston, Massachusetts USA) were used for statistical analysis. Descriptive statistics were used to calculate the proportion of positive neurodynamic tests. This was expressed as number and percentage of participants with positive and negative neurodynamic tests. The association between nerve mechanosensitivity and DPN severity was explored by using the Chi square test for independence [36]. The Shapiro Wilk was used to assess if the data was normally distributed. The difference in TCSS score between positive and negative SLR and slump tests was calculated by T test or its non-parametric equivalent [36].

## 3. Results

### 3.1. Demographics

A total of 90 individuals were assessed for eligibility. Forty-seven individuals either declined to participate or were excluded from the study due to presence of back and/or spinally referred leg pain, metabolic and/or neurological disease, as well as, due to presence of subclinical diabetic neuropathy. Forty-three participants with T1DM and DPN took part in the present study. Thirty-nine participants had confirmed DPN diagnosis. The remaining 4 did not have a confirmed DPN diagnosis at the time of study but had DPN related symptoms and scored 6 points (or greater) on TCSS suggesting ‘mild neuropathy.’

Most participants (32) were recruited via the diabetes department’s outpatient list. The remaining 11 participants were recruited via invitation posts on social media and e-mail recruitments. Twenty-two male and 21 female participants were recruited, with mean age of 38.2 years (Table 1).

**Table 1.** Participants’ demographics.

Demographics	Mean±Standard deviation
Age (years)	38.2±9.6
Sex assigned at birth	22Male 21Female
BMI	24.8±2.6
T1DM duration (years)	23.9±2.3
DPN duration (years)	4.9±3.57

Abbreviations: BMI: Body mass index; T1DM: Type 1 diabetes mellitus; DPN: Diabetic peripheral neuropathy.

In addition, TCSS descriptive statistics for positive and negative SLR and slump tests are detailed in Table 2.

**Table 2.** Descriptive statistics for TCSS scores for neurodynamic tests.

TCSS (Outcome of neurodynamic tests)	Median, Interquartile range
TCSS (positive SLR) TCSS (negative SLR) TCSS (positive slump) TCSS (negative slump)	7, 7 14, 7 7, 7 14, 7

Abbreviations: TCSS: Toronto clinical scoring system; SLR: Straight leg raise.

### 3.2. Proportion of positive and negative straight leg raise and slump test

The SLR test was positive in 20 participants (46%) (95% confidence intervals (CI) 46%–61%) and negative in 23 participants (54%) (95%CI 54%–67%). Specific to the slump test, 24 participants (56%) (95% CI 56%–69%) had a positive test, while 19 participants (44%) (95% CI 44%–58%) had a negative test.

### 3.3. Association between straight leg raise test outcomes and diabetic peripheral neuropathy severity using TCSS score

#### 3.3.1. Positive SLR test

**Figure 1.**
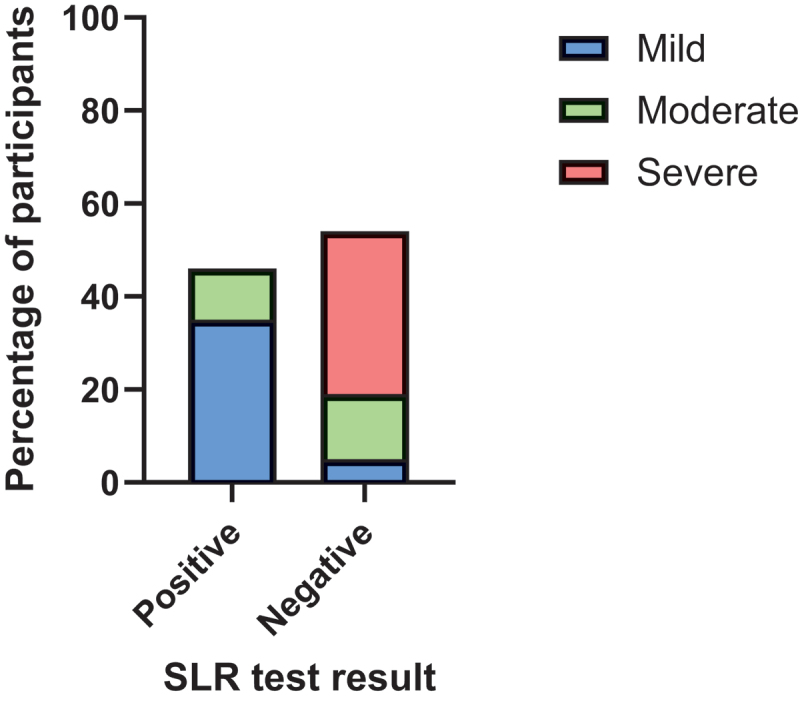
Proportion of participants with positive/negative SLR test and mild, moderate and severe DPN, measured using TCSS.

Abbreviations: SLR: Straight leg raise; DPN:Diabetic peripheral neuropathy; TCSS: Toronto clinical scoring system.

Fifteen participants (35%) had mild DPN, 5 (11%) had moderate and none of the participants with severe DPN had a positive SLR test (Figure 1).

#### 3.3.2. Negative SLR test

Two participants (5%) had mild DPN, 6 (14%) moderate and 15 (35%) had severe DPN with a negative SLR test (Figure 1).

There was a statistically significant relationship between SLR test result and DPN severity (Pearson chi square 57.59, *p* < 0.0001).

### 3.4. Association between slump test outcomes and diabetic peripheral neuropathy severity using Toronto score

#### 3.4.1. Positive slump test

**Figure 2.**
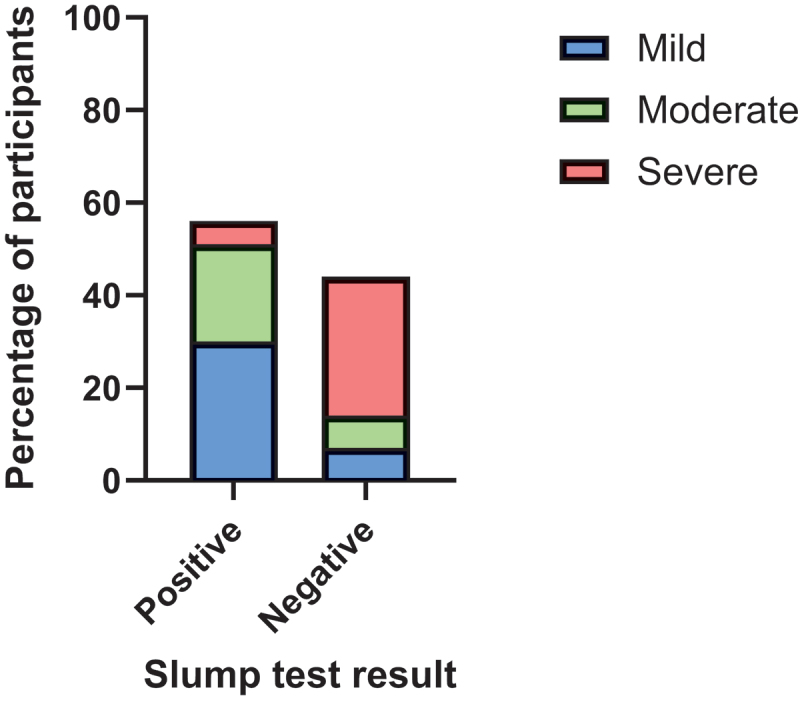
Proportion of participants with positive/negative slump test and mild, moderate and severe DPN, measured using TCSS.

Abbreviations: DPN: Diabetic peripheral neuropathy; TCSS: Toronto Clinical Scoring System.

Thirteen participants (30%) had mild DPN, 9 (21%) moderate and 2 (5%) had severe DPN with a positive slump test (Figure 2).

#### 3.4.2. Negative slump test

Three participants (7%) had mild DPN, 3 (7%) moderate and 13 (30%) had severe DPN with a negative slump test (Figure 2).

There was a statistically significant relationship between slump test result and DPN severity (Pearson chi square 38.27, *p* < 0.0001).

### 3.5. TCSS score in participants with positive and negative neurodynamic test

The TCSS data was not normally distributed (Shapiro Wilk test < 0.05) therefore Mann Whitney tests were used to determine the differences in TCSS scores between participants with a positive and a negative neurodynamic test.

#### 3.5.1. SLR test

**Figure 3.**
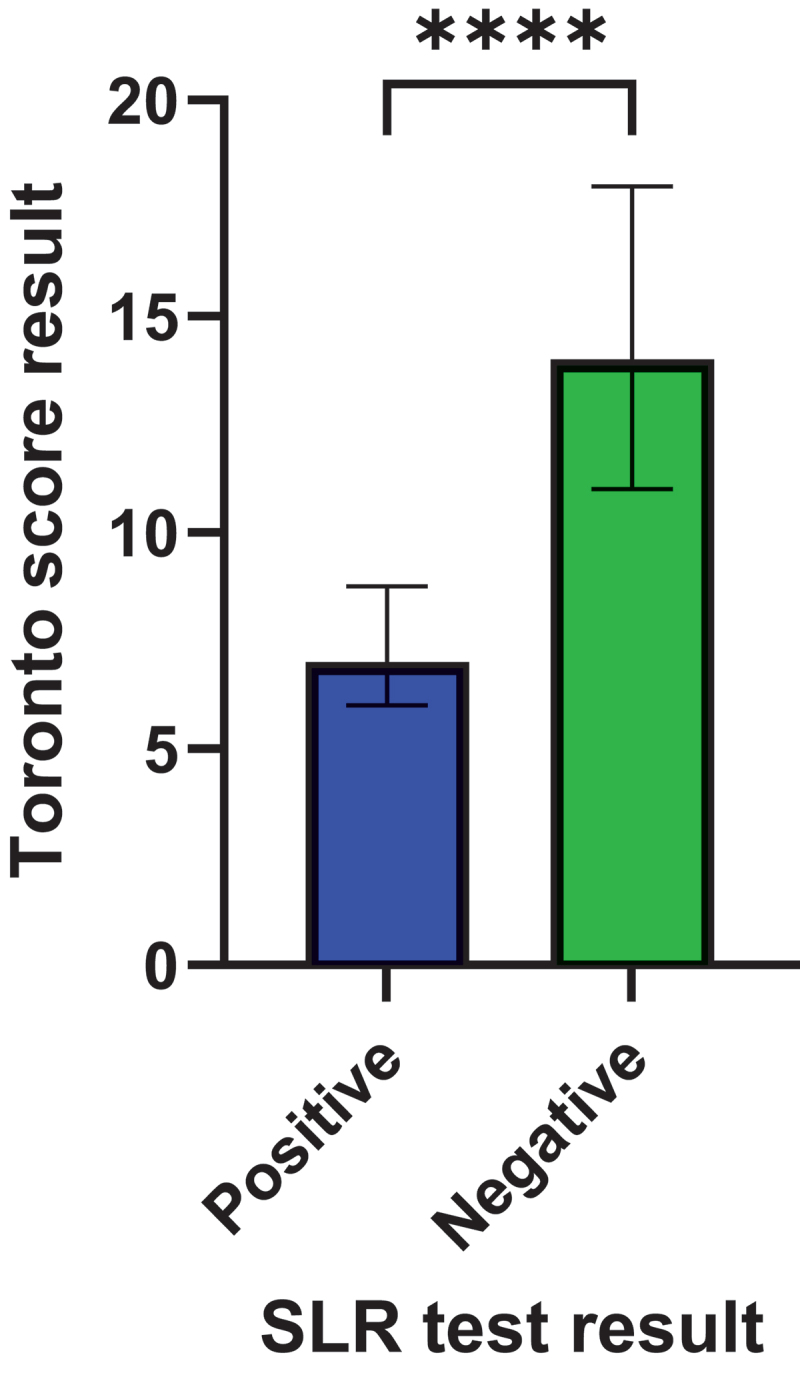
Difference on TCSS score between participants with positive and negative SLR test. Error bars indicate the interquartile range. A higher TCSS score indicates more severe neuropathy.

Abbreviations: SLR: Straight leg raise; DPN:Diabetic peripheral neuropathy; TCSS: Toronto Clinical Scoring System.

Participants with negative SLR test had significantly higher TCSS scores than participants with positive SLR test (*p* < 0.0001) (Figure 3).

#### 3.5.2. Slump test

**Figure 4.**
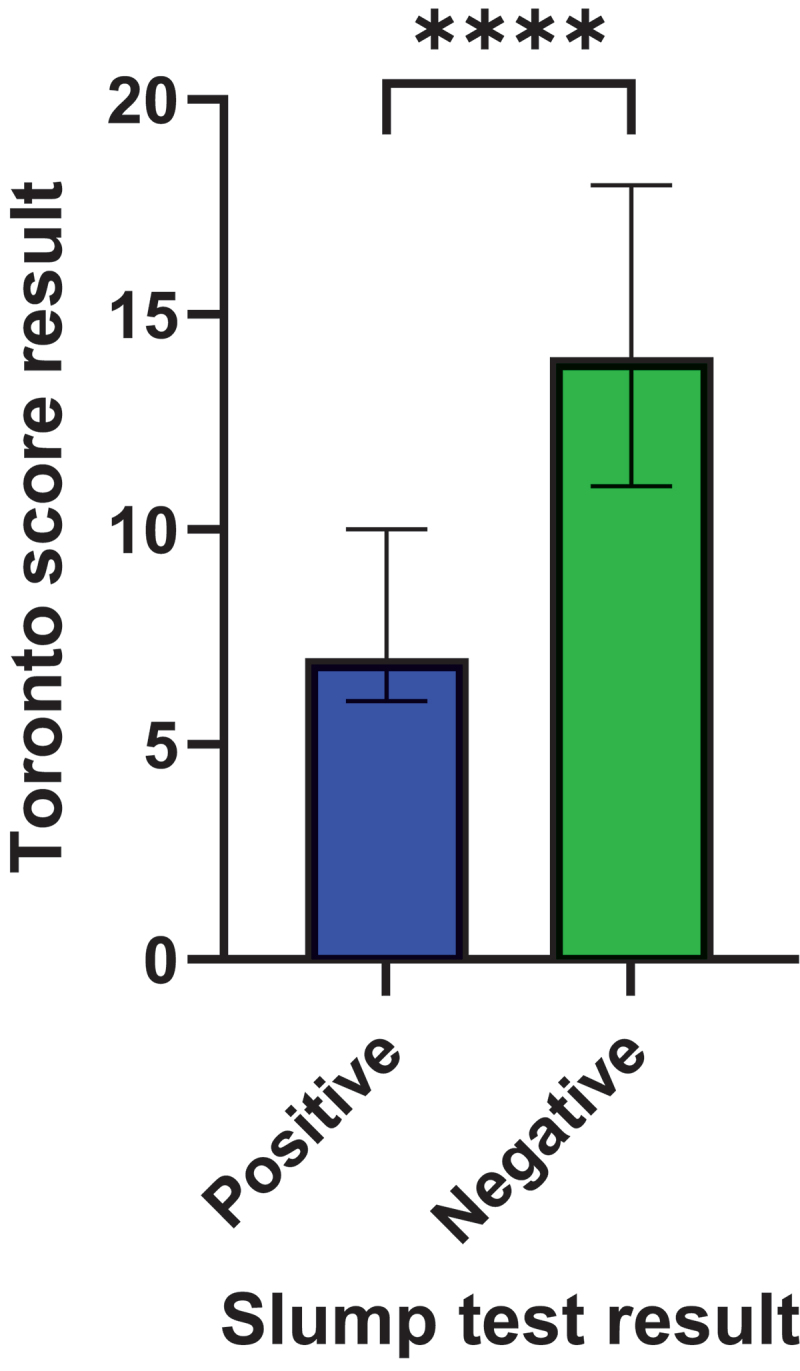
Difference on TCSS score between participants with positive and negative slump test. Error bars indicate the interquartile range. A higher TCSS score indicates more severe neuropathy.

Abbreviations: DPN: Diabetic peripheral neuropathy; TCSS: Toronto Clinical Scoring System.

Participants with a negative slump test had significantly higher TCSS scores than individuals with positive slump test (*p* < 0.0001) (Figure 4).

## 4. Discussion

To the authors' knowledge, this is the first study that has determined the proportion of people with T1DM and DPN with heightened nerve mechanosensitivity, as assessed with SLR and slump tests. Additionally, the study uniquely highlights the effect DPN severity has on heightened nerve mechanosensitivity. A greater proportion of people with a negative SLR and a negative slump test had more severe DPN, as assessed by having a TCSS > 13 points, than participants with a positive test. Individuals with a negative SLR and a negative Slump test scored higher on TCSS, suggesting a more severe DPN, compared to individuals with a positive SLR and a positive Slump test.

### 4.1. Proportion of people with heightened nerve mechanosensitivity

Straight leg raise and slump tests were positive in almost 50% and 60% of participants, respectively. This indicates that heightened nerve mechanosensitivity is present in around half of people with DPN. A notable proportion of people with T1DM and DPN had negative neurodynamic tests. There was a greater proportion of positive slump tests in the participants which may be explained by the addition of trunk and cervical flexion during the test contributing greater amounts of tensile load on the nervous system [37]. It may be that a greater challenge on the nervous system is more likely to produce a heightened nerve response in individuals with T1DM and DPN.

In a previous study [28], individuals with type 2 diabetes mellitus and DPN had a reduction in the range of hip flexion with the addition of sensitizing maneuvers at the ankle during the SLR test movements that increase the tensile load applied to the targeted nerve [24]. Since mechanosensitivity is a normal response to the mechanical load applied to the nerve [12–14], a reduction in range of hip flexion with the application of sensitizing ankle maneuvers in the less severe DPN group suggests a heightened nerve mechanosensitivity response [28]. As this response is also seen in individuals without nerve pathology [12–14] a comparison to a healthy control group is necessary. The average restriction in range was greater than previously reported restrictions in healthy control participants [13], suggesting heightened nerve mechanosensitivity. However, the lack of direct comparison to a healthy control cohort in the Boyd et al [28] study might make these conclusions less certain.

### 4.2. Severity of DPN affects heightened nerve mechanosensitivity

Negative SLR and slump tests were associated with a more severe DPN presentation. This was supported by the significantly higher TCSS scores in participants with negative SLR and slump tests. Individuals with mild and moderate DPN and lower TCSS scores demonstrated both positive and negative SLR and slump tests. Since a higher TCSS score suggests a more severe DPN [11], these findings indicate that heightened nerve mechanosensitivity is reduced in people with T1DM and severe DPN presentation.

The findings of the present study are in line with Boyd et al. [28] in participants with severe DPN from type 2 diabetes mellitus. In this study, individuals with severe DPN demonstrated little change to their range of hip flexion during the SLR when a sensitizing maneuver at the ankle was applied. This suggests reduced nerve mechanosensitivity compared to the less severe DPN group which demonstrated a reduction in the range of hip flexion following application of ankle sensitizing maneuvers [28]. Although the outcome measures in our study were different from Boyd et al [28] study which looked at hip ROM, both studies suggest that heightened nerve mechanosensitivity is affected by the degree of DPN severity in individuals with type 1 and type 2 diabetes.

At present, it is not known why people with T1DM and severe DPN have reduced heightened nerve mechanosensitivity. However, pathophysiological mechanisms of DPN might give an insight into this observation. In DPN, there is a progressive distal to proximal axonal degeneration which gradually leads to loss of function of both large and small diameter axons [7]. As a result, the initial demyelination and degeneration of nerve axons that occurs leads to up-regulation and accumulation of ion channels [8]. In severe DPN where nerve axons have sufficiently degenerated [7,38], heightened nerve mechanosensitivity may be absent since the nociceptive afferents have reduced their firing in response to mechanical loading. Interestingly, in a cohort of participants with carpal tunnel syndrome and diminished warm detection thresholds, reduced responses to upper limb neurodynamic tests were found [19]. This was attributed to a decrease in activity of nociceptive C fibers that innervate the nervi nervorum of the connective tissue of the nerves of the upper limb [19].

### 4.3. Limitations

In the present study, most participants had confirmed DPN diagnosis via nerve conduction studies in addition to bilateral symmetrical lower limb neuropathy symptoms [3]. However, 4 participants did not undergo electrodiagnostic studies at the time of the study. Abnormal findings on those studies are essential for a confirmed DPN diagnosis [3]. Thus, the presence and grading of DPN in those participants was assessed according to TCSS. Due to moderate, significant correlation of TCSS screening tool with the gold standard of myelinated fiber density [11], it is possible that these participants might have been misclassified as having DPN.

The same examiner performed the DPN examination according to the TCSS and the neurodynamic assessment. This might have caused some degree of bias in the study.

In our study, we acknowledge that the sample size was smaller than what our initial sample size calculation suggested. Despite this, we observed statistically significant differences between our two groups suggesting that the effect size was large enough to be detected, indicating a meaningful difference between the groups.

### 4.4. Clinical implications

Heightened nerve mechanosensitivity has been observed during lower limb neurodynamic assessment, in people with T1DM and less severe DPN. However, as many individuals with severe DPN are likely to demonstrate negative SLR and slump tests in this study, lower limb neurodynamic tests should not be used to determine the presence of neuropathy, in individuals with T1DM.

## 5. Conclusion

Up to 60% of individuals with T1DM and DPN had positive lower limb neurodynamic tests. Most participants with T1DM and severe DPN, as indicated by high scores on TCSS, had negative lower limb neurodynamic tests. Similar to other studies in type 2 diabetes mellitus and carpal tunnel syndrome, this suggests that people with more severe neuropathy have reduced nerve mechanosensitivity. Future studies are required using a larger sample to confirm our findings regarding the association between heightened nerve mechanosensitivity and the extent of loss of sensory nerve
